# Dental Health in Pediatric Patients With Different Types of Mucopolysaccharidosis: Retrospective Cross-Sectional Study

**DOI:** 10.2196/87430

**Published:** 2026-05-04

**Authors:** Mengxing Wang, Rong Liu, Tian Xia, Ying Wang

**Affiliations:** 1 Department of Stomatology, Capital Center for Children’s Health, Capital Medical University, No. 2 Yabao Road, Chaoyang District, Beijing, 100020, China, 86 13681057972; 2 Department of Hematology, Capital Center for Children’s Health, Capital Medical University, Beijing, China

**Keywords:** mucopolysaccharidosis, dental caries, dental anomalies, pediatrics, retrospective

## Abstract

**Background:**

Patients with mucopolysaccharidosis (MPS) appear to have an increased risk of developing dental disease.

**Objective:**

This study aimed to evaluate the status of dental caries and dental anomalies among Chinese patients with different types of MPS.

**Methods:**

This retrospective study analyzed a consecutive cohort of 102 pediatric patients with MPS who visited the Department of Stomatology at the Capital Center for Children’s Health between August 2010 and August 2025. Eligible patients were defined as those with a confirmed diagnosis of MPS who were aged ≤14 years at the time of their dental visit and had complete dental examination records available. Dental caries and anomalies were assessed through clinical records and radiographic data.

**Results:**

Dental caries were observed in 55.9% (57/102) of patients, and no statistically significant difference was observed across the MPS subtypes (*P*=.72). Deep dentinal caries (d4-6mft) were observed in 40.2% (41/102) of the participants and contributed most to the total decayed, missing, and filled teeth index score. The overall prevalence of dental anomalies was 32.4% (33/102), with a statistically significant difference among MPS subtypes (*P*=.005). Patients with MPS type IV had a significantly higher risk of dental anomalies compared to those with MPS type II (odds ratio 6.32, 95% CI 1.55‐28.28; *P*=.01), after adjusting for age and gender.

**Conclusions:**

The prevalence of dental anomalies differed significantly across MPS subtypes, while that of dental caries did not. These findings emphasize the need for early, targeted preventive care and tailored dental interventions to improve oral health outcomes in this population.

## Introduction

Mucopolysaccharidosis (MPS) is a group of inherited conditions involving metabolic dysfunction. Lysosomal enzyme deficiency leads to the accumulation of glycosaminoglycans (GAGs), resulting in systemic symptoms, and is categorized into 7 primary types (I [Hurler], II [Hunter], III [Sanfilippo], IV [Morquio], VI [Maroteaux-Lamy], VII [Sly], IX [Natowicz]) caused by the deficiency of 1 of 11 different enzymes. The inability to break down GAGs leads to their abnormal accumulation within various tissues and cells, progressively resulting in cellular dysfunction [1,2]. Globally, the estimated prevalence ranges from 1.04 to 4.8 per 100,000 live births [3-5]. In China, a study conducted across 21 provinces and municipalities between 2006 and 2012 reported that among 376 diagnosed cases of lysosomal storage diseases, MPS accounted for 50.5% (n=190 cases), with MPS type II (MPS-II) being the most common subtype, comprising 47.4% of all MPS cases [6].

Dental and craniofacial abnormalities were frequently reported in patients with MPS [7,8]. Common oral manifestations included macroglossia, anterior open bite, delayed eruption of teeth, cystic lesions, and temporomandibular joint abnormalities [9,10]. These patients also exhibited a higher susceptibility to dental caries, underscoring the need for preventive oral health measures. Meanwhile, due to the low prevalence of MPS and the fact that oral health issues are not among the life-threatening manifestations of the disease, patients rarely seek dental care voluntarily, which explains the current paucity of research on dental caries in this specific population. While previous studies on dental health in patients with MPS have primarily consisted of case reports, small case series, and small cohort studies, the overall oral health profile of these patients is poorly characterized.

Furthermore, MPS encompasses 7 distinct clinical subtypes, each exhibiting significant heterogeneity in systemic manifestations due to specific enzymatic deficiencies and the resultant accumulation of various GAGs (eg, dermatan sulfate, heparan sulfate, keratan sulfate, chondroitin sulfate, or hyaluronan) [11-14]. This systemic variability is likely reflected in oral health. For instance, the severe skeletal dysplasia, particularly prominent in MPS-IV, can lead to distinct craniofacial patterns, malocclusions, and potential impacts on tooth eruption and morphology [15]. Additionally, a Brazilian study involving 17 patients with either MPS-IV or VI found that enamel hypoplasia was observed exclusively in those with MPS-IV, while anterior open bite was present only in patients with MPS-VI [16]. Similarly, variations in the degree of soft tissue hyperplasia and the patient’s capacity to maintain oral hygiene across different subtypes may influence caries risk. A UK single-center study reported a higher incidence of dental caries in patients with MPS-IV compared to the general population and other MPS groups [17]. Despite these plausible variations and growing international data, comprehensive and comparative analyses of oral phenotypes among MPS subtypes, particularly within the Chinese population, remain limited.

Therefore, based on electronic medical record data, this study investigated the prevalence of dental caries and dental anomalies among patients with different MPS subtypes and analyzed the correlation between dental anomalies and caries in patients with MPS to provide insights for improving preventive care and treatment strategies.

## Methods

### Ethical Considerations

This study was conducted in accordance with the Strengthening the Reporting of Observational Studies in Epidemiology (STROBE) statement. The study was approved by the ethics review board of Capital Institute of Pediatrics (SHERLLM2024031) and was registered with the Chinese Clinical Trial Registry (ChiCTR2400090276). All study procedures were in strict compliance with the principles of the Declaration of Helsinki, and written informed consent was waived due to the retrospective nature of the study. To protect patient privacy, all data were anonymized and deidentified prior to analysis. Access to the original medical records was restricted to the research team, and no personally identifiable information (eg, names, medical record numbers, or exact dates) was extracted or reported.

### Patients and Study Design

This retrospective cross-sectional study included pediatric patients diagnosed with MPS who visited the Department of Stomatology at the Capital Center for Children’s Health between August 2010 and August 2025. This study used a convenience sampling method based on consecutive cases. The inclusion criteria were as follows: (1) a confirmed diagnosis of any subtype of MPS based on enzymatic or genetic testing, (2) age ≤14 years, and (3) availability of a complete dental examination record. Patients were excluded if they had concurrent major systemic conditions (unrelated to MPS) that could severely confound oral health status, such as uncontrolled diabetes, severe congenital heart disease, autoimmune diseases with oral manifestations, or a history of radiotherapy to the head and neck region. On the basis of these criteria, a total of 102 eligible patients were included in the final analysis.

The retrospective collection of electronic medical records was initiated after the study was registered on September 29, 2024. For patients who had multiple visits, only data from their first visit were included in the analysis. It should be noted that the hematology department of our single-center hospital is the largest MPS center in the country, and most patients visiting the stomatology department were referred for routine pretransplantation check-ups prior to stem cell transplantation, rather than presenting with specific oral problems. Therefore, although the data were derived from the stomatology department, the primary purpose of the patients’ visits was stem cell transplantation, not specifically for oral issues. This indicates that the results of the oral examinations do not solely reflect the oral health of patients who actively seek treatment for oral problems in the stomatology department, thereby reducing the likelihood of selection bias to some extent. The findings of this study are more representative of the oral health status of patients with MPS, rather than being confined to those with severe oral problems.

### Data Collection

The dental conditions of these patients were assessed by reviewing historical clinical records and radiographic data. Data were systematically extracted from oral health records, including demographic information (eg, age and gender), MPS subtype classifications, and comprehensive dental evaluations.

Dental anomalies were categorized into developmental (delayed or retained eruption, delayed tooth germ formation, delayed tooth development, and prolonged retention of primary teeth), morphological (conoid teeth, taurodontism, enamel hypoplasia, and microdontia), numerical (hypodontia and supernumerary teeth), positional (ectopic eruption, impaction, diastema, tooth inversion, and migration), and occlusal abnormalities (biprotrusion, crossbite, crowding, and spaced arches) [8]. Root developmental features (delayed formation, dilaceration, and elongated roots) and periodontal conditions (bone rarefaction and radiolucent lesions) were documented alongside dental follicle abnormalities.

Caries assessment used standardized diagnostic criteria through the International Caries Detection and Assessment System (ICDAS) [18] and the decayed, missing, and filled teeth index (dmft) [19]. All ICDAS codes were assigned based on standardized clinical intraoral visual examination. Lesion characteristics were recorded in terms of frequency (per-arch counts), anatomical distribution (maxillary or mandibular and anterior or posterior), and progression depth (ICDAS scores 1‐6). All clinical examinations were performed by attending dentists as part of routine patient care over the study period. Consequently, multiple examiners were involved. While formal, prospective interexaminer calibration specific to this study was not feasible due to its retrospective design, the consistent use of the ICDAS and dmft criteria across the department provided a standardized framework for diagnosis and recording. In addition, a single primary investigator reviewed all original clinical notes. Any ambiguous, inconsistent, or missing entries were coded as “data missing” rather than inferred to avoid interpretation bias.

### Statistical Analysis

All statistical analyses were performed using R software (version 4.3.2; R Foundation for Statistical Computing). Continuous variables were described using mean (SD), while categorical variables were summarized using frequency (percentage). The normality of continuous variables was assessed using the Shapiro-Wilk test. Differences in the prevalence of dental caries and anomalies among MPS subtypes were analyzed with the chi-square or Fisher exact test. The Kruskal-Wallis test was used to compare the dmft index across subtypes. Where these tests revealed a statistically significant difference, post hoc pairwise comparisons were carried out to identify the specific pairs of subtypes that differed. Multivariable logistic regression was employed to evaluate the association between different MPS subtypes and dental health after adjusting for age and gender. Odds ratios (ORs) with 95% CIs were calculated to quantify effect sizes. A *P* value of less than .05 was considered statistically significant.

## Results

### Participant Characteristics

**Figure 1.**
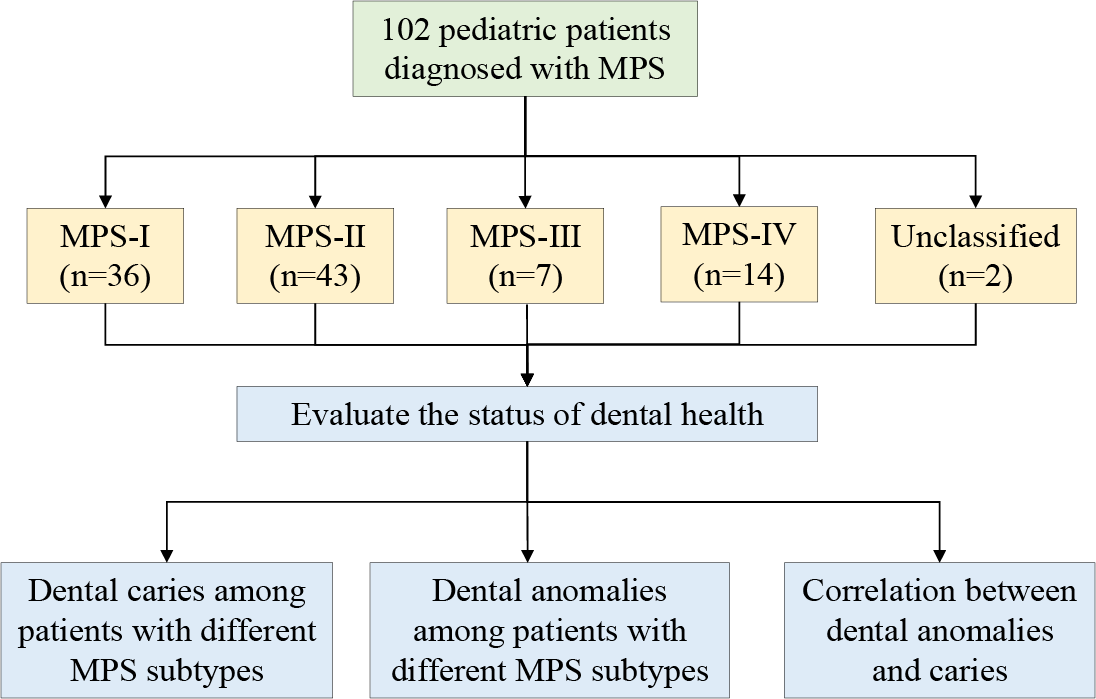
Research framework diagram. MPS: mucopolysaccharidosis.

A total of 102 patients with MPS were included in this study (Figure 1), and their baseline demographic and clinical characteristics are presented in Table 1. Of these, 73.5% (75/102) were male, with a mean age of 4.8 (SD 2.6) years, ranging from 1 to 12.9 years. Regarding the age at diagnosis, 42.2% (43/102) of patients were aged 3 years or younger, 36.3% (37/102) were aged between 4 and 6 years, and 21.6% (22/102) were aged 7 years or older. MPS-II was the most common subtype (43/102, 42.2%), followed by MPS-I (36/102, 35.3%), while MPS-IV and MPS-III accounted for 13.7% (14/102) and 6.9% (7/102), respectively. Two cases (2/102, 2%) were unclassified. In terms of oral health status, the mean dmft index of the cohort was 2.7 (SD 3.4), with dental caries experience observed in 55.9% (57/102) of patients. Dental anomalies were detected in 32.4% (33/102) of the patients.

**Table 1.** Demographic and clinical characteristics of patients with mucopolysaccharidosis (MPS; N=102).

Characteristics	Values
Male, n (%)	75 (73.5)
Age (years), mean (SD; range)	4.8 (2.6; 1‐12.9)
Age at diagnosis (years), n (%)	
≤3	43 (42.2)
4-6	37 (36.3)
≥7	22 (21.6)
MPS type, n (%)	
MPS-I	36 (35.3)
MPS-II	43 (42.2)
MPS-III	7 (6.9)
MPS-IV	14 (13.7)
Unclassified	2 (2)
Dental caries, n (%)	57 (55.9)
dmft^a^ index, mean (SD)	2.7 (3.4)
Dental anomalies, n (%)	33 (32.4)

a dmft: decayed, missing, and filled teeth.

### MPS Subtypes and Dental Caries

Table 2 presents the detailed characteristics of dental caries among patients with different MPS subtypes. There was no statistically significant difference observed across the MPS subtypes in dental caries (*P*=.72). Specifically, caries prevalence was 58.3% (21/36) in MPS-I, 51.2% (22/43) in MPS-II, 71.4% (5/7) in MPS-III, and 50% (7/14) in MPS-IV.

A total of 279 carious teeth were recorded. Patients with MPS-II had the highest number of carious teeth, followed by MPS-I, MPS-IV, and MPS-III. The majority of carious teeth were deciduous across all groups. By tooth type, molars were the most commonly affected, particularly in MPS-IV (35 carious molars among 7 patients) and MPS-II (66 carious molars among 22 patients). Carious lesions were more frequently observed in the maxillary teeth than in the mandibular teeth in all groups except MPS-IV.

For the dmft index, MPS-II showed the highest value (mean 5.68, SD 3.47), while MPS-III had the lowest (mean 2.40, SD 2.51). No statistically significant difference was observed among the 4 subtypes (*P*=.15). Further analysis of caries severity showed that the d4-6mft (deep dentinal lesions) were observed in 40.2% (41/102) of the participants and contributed the most to the total dmft score. Molar teeth had the highest mean dmft values in patients with MPS-IV (mean 5.00, SD 2.45), whereas patients with MPS-II exhibited the highest caries experience in maxillary teeth (mean 3.55, SD 1.99).

**Table 2.** Dental caries in patients with different types of mucopolysaccharidosis (MPS).

Characteristics	MPS-I (n=36)	MPS-II (n=43)	MPS-III (n=7)	MPS-IV (n=14)	Unclassified (n=2)	*P* value^a^
Dental caries, n (%)	21 (58.3)	22 (51.2)	5 (71.4)	7 (50)	2 (100)	.72
Carious teeth, n^b^	94	126	24	37	5	—^c^
Carious deciduous teeth, n	94	125	19	37	5	—
Carious incisors	37	58	5	2	4	
Carious canines	5	1	1	0	0	
Carious molars	52	66	13	35	1	
Carious maxillary teeth	58	78	13	16	4	
Carious mandibular teeth	36	47	6	21	1	
dmft^d^ index (n=57), mean (SD)	4.48 (3.14)	5.68 (3.47)	2.40 (2.51)	5.29 (2.56)	2.50 (2.12)	.15
d1-2mft	0.43 (1.21)	0.55 (1.26)	0 (0)	0 (0)	—	
d3mft	0.67 (1.85)	0.86 (1.55)	0.20 (0.45)	1.86 (1.46)	—	
d4-6mft	3.38 (3.34)	4.27 (3.65)	2.20 (2.68)	3.43 (3.31)	—	
Carious incisors dmft	1.76 (1.87)	2.64 (1.73)	0.60 (0.89)	0.29 (0.49)	—	
Carious canines dmft	0.24 (0.77)	0.05 (0.21)	0.20 (0.45)	0 (0)	—	
Carious molars dmft	2.43 (2.42)	3.09 (2.78)	2.60 (3.44)	5.00 (2.45)	—	
Carious maxillary teeth dmft	2.76 (2.30)	3.55 (1.99)	2.60 (2.41)	2.29 (1.89)	—	
Carious mandibular teeth dmft	1.71 (1.65)	2.55 (2.40)	1.20 (1.64)	3.00 (1.15)	—	

a Compared the differences among the 4 groups: MPS-I, MPS-II, MPS-III, and MPS-IV.

b Values represent cumulative counts of carious teeth. Percentages were not calculated because total tooth counts per group were not available*.*

c Not applicable*.*

d dmft: decayed, missing, and filled teeth.

### MPS Subtypes and Dental Anomalies

Table 3 summarizes the prevalence and types of dental anomalies across different MPS subtypes. The overall prevalence of dental anomalies was 32.4% (33/102), with a statistically significant difference among MPS subtypes (*P*=.005). A post hoc analysis revealed a statistically significant difference between MPS-II and MPS-IV (*P*=.007). Patients with MPS-IV exhibited the highest prevalence of dental anomalies (9/14, 64.3%), followed by MPS-I (15/36, 41.7%), MPS-III (2/7, 28.6%), and MPS-II (7/43, 16.3%). No anomalies were detected in the 2 (100%) patients with unclassified MPS.

**Table 3.** Dental anomalies in patients with different types of mucopolysaccharidosis (MPS).

Characteristics	MPS-I (n=36), n (%)	MPS-II (n=43), n (%)	MPS-III (n=7), n (%)	MPS-IV (n=14), n (%)	Unclassified (n=2), n (%)	*P* value^a^
Total anomalies	15 (41.7)	7 (16.3)	2 (28.6)	9 (64.3)	0 (0)	.005
Developmental anomalies	9 (25)	3 (7)	1 (14.3)	2 (14.3)	0 (0)	—^b^
Morphological anomalies	3 (8.3)	3 (7)	0 (0)	4 (28.6)	0 (0)	—

a Compared the differences among the 4 groups: MPS-I, MPS-II, MPS-III, and MPS-IV.

b Not applicable.

Developmental anomalies were the most common type observed in patients with MPS-I (9/36, 25%), while morphological anomalies were notably frequent in patients with MPS-IV (4/14, 28.6%). Patients with developmental anomalies primarily exhibited the following 4 manifestations: delayed or retained eruption, delayed tooth germ formation, delayed tooth development, and prolonged retention of primary teeth. Among these, delayed tooth germ formation occurred exclusively in patients with MPS-II, and delayed tooth development was only observed in patients with MPS-I and MPS-II. Only one 9-year-old patient (2.8%) with MPS-I presented with 3 types of developmental anomalies simultaneously, and all other patients (97.2%) exhibited only 1 type. Among patients with morphological anomalies, one 9-year-old patient with MPS-IV (14.3%) exhibited conoid teeth in 2 maxillary permanent molars, while all other cases (85.7%) presented with enamel hypoplasia.

Numerical anomalies were observed only in patients with MPS-III (1/7, 14.3%) and MPS-IV (1/14, 7.1%). All patients with numerical anomalies exhibited missing teeth. Positional anomalies were present in patients with MPS-I (3/36, 8.3%) and MPS-III (1/7, 14.3%), whereas root anomalies were rare and detected in only 1 (2.3%) patient with MPS-II. Among all patients with positional anomalies, one case presented with an anterior crossbite, while the others exhibited malposition of unerupted teeth. The patient with root anomalies (MPS-II) showed root dilaceration. Bone anomalies were most prevalent in patients with MPS-I (7/36, 19.4%), and follicle anomalies were exclusively found in patients with MPS-I (2/36, 5.6%) and MPS-II (2/43, 4.7%). Patients with bone anomalies primarily presented with radiolucent bone lesions and condylar resorption. Those with follicle anomalies mainly exhibited large dental follicles.

### Multivariable Analysis of the Association of MPS Subtypes With Dental Health

**Figure 2.**
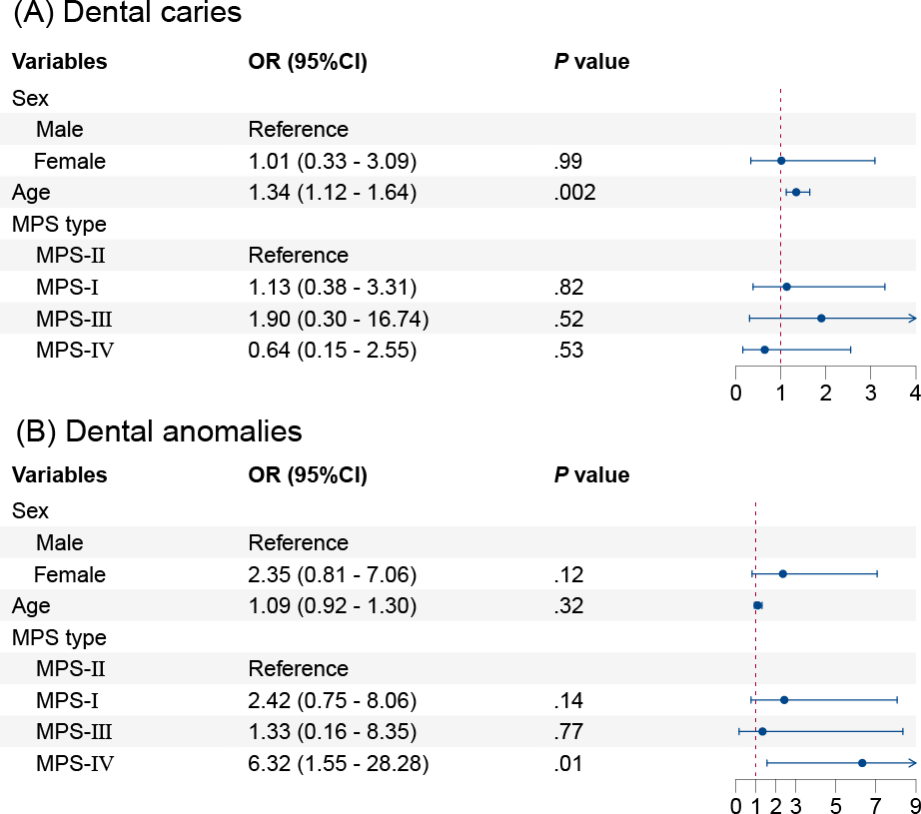
Multivariable analysis of the association of mucopolysaccharidosis (MPS) subtypes with dental health: (A) dental caries and (B) dental anomalies. OR: odds ratio.

Multivariable logistic regression analysis was used to evaluate the association between MPS subtypes and dental health outcomes after adjusting for sex and age. Patients with MPS-II were the most prevalent and were therefore designated as the reference group. No significant association was observed between MPS subtypes and dental caries. In contrast, patients with MPS-IV had a significantly higher risk of dental anomalies compared to those with MPS-II (OR 6.32, 95% CI 1.55‐28.28; *P*=.01; Figure 2).

### Relationship Between Dental Anomalies and Dental Caries in Patients With MPS

The relationship between dental anomalies and dental caries in patients with MPS is presented in Table 4. No significant difference was observed in the prevalence of total dental anomalies between patients with and without caries (33.3% vs 31.1%; *P*=.81). Similarly, the presence of developmental anomalies was not significantly associated with dental caries (15.8% vs 13.3%; *P*=.73).

However, morphological anomalies were significantly less frequent in patients with dental caries compared to those without (3.5% vs 17.8%; *P*=.04), with an OR of 0.17 (95% CI 0.02‐0.72). In contrast, bone anomalies were significantly more common among patients with caries (19.3% vs 2.2%; *P*=.008). The odds of having dental caries were approximately 10.5 times higher in patients with bone anomalies (95% CI 1.3‐84.93).

**Table 4.** Relationship between dental anomalies and dental caries in patients with mucopolysaccharidosis.

Dental anomalies	Without dental caries (n=45), n (%)	With dental caries (n=57), n (%)	*P* value	OR^a^ (95% CI)
Total anomalies	14 (31.1)	19 (33.3)	.81	1.11 (0.48‐2.56)
Developmental anomalies	6 (13.3)	9 (15.8)	.73	1.22 (0.4‐3.91)
Morphological anomalies	8 (17.8)	2 (3.5)	.04	0.17 (0.02‐0.72)
Bone anomalies	1 (2.2)	11 (19.3)	.008	10.52 (1.3‐84.93)

a OR: odds ratio.

## Discussion

This retrospective analysis of 102 pediatric patients with MPS in China revealed distinct oral health characteristics across MPS subtypes. The primary findings include an overall dental caries prevalence of 55.9% (57/102), with no significant differences among subtypes (*P*=.72). In contrast, dental anomalies were observed in 32.4% (33/102) of patients, showing significant subtype-specific variation (*P*=.005), with the highest prevalence in MPS-IV (9/14, 64.3%). Further analysis indicated that deep dentinal lesions (d4-6mft) contributed the most to the total dmft score, and molars were the most frequently affected teeth.

These findings are significant because they systematically characterize oral phenotypic variability among MPS subtypes in a Chinese pediatric population for the first time. The subtype-specific distribution of dental anomalies suggests that the underlying pathophysiology of MPS may differentially affect odontogenesis, while the relatively uniform caries prevalence indicates that common risk factors, such as impaired oral hygiene maintenance, may transcend subtype-specific differences. This understanding can assist clinicians in developing tailored oral health management strategies for different MPS subtypes.

Our findings align with and extend the existing body of evidence regarding oral manifestations in patients with MPS. The overall dental caries prevalence of 55.9% (57/102) observed in our cohort corresponds with previous reports of elevated caries experience in this population. For instance, a Brazilian cross-sectional study documented significantly higher caries rates among patients with MPS relative to unaffected individuals [20,21], while a study of Indian children reported increased caries indices in both deciduous and permanent dentitions in the MPS group compared to healthy controls [22]. Similarly, individuals with rare genetic disorders, including MPS, have been shown to possess a substantially elevated likelihood of developing dental caries [23]. Notably, our observation of a predominance of deep dentinal caries adds granularity to these earlier reports, suggesting that MPS-specific factors—such as enamel structural alterations, restricted mouth opening, and functional limitations—may predispose patients to more advanced carious lesions.

Regarding dental anomalies, our results corroborate the established understanding that such abnormalities are a frequent component of the MPS phenotype. Carneiro et al [8] highlighted a range of common radiographic findings in patients with MPS, including supernumerary teeth, peg-shaped teeth, taurodontism, tooth impaction, and dilated dental roots. This is consistent with the spectrum of anomalies identified in our cohort. Further supporting this pattern, Almeida-Barros et al [16] reported supernumerary teeth in 23.5% of patients with MPS-IV and MPS-VI, and another cross-sectional study noted a high frequency of cyst-like dental crypts, taurodontism, and tooth impaction among patients with MPS [21]. The convergence of these international findings underscores that dental anomalies are a core, recurrent feature across diverse MPS populations, likely stemming from the underlying disruption of craniofacial and dental development by GAG accumulation.

A key contribution of our study lies in the comparative analysis across MPS subtypes. We found no statistically significant difference in caries prevalence or the dmft index among subtypes, suggesting that universal risk factors, such as the profound challenges in maintaining oral hygiene common to all patients with MPS, may outweigh potential subtype-specific pathophysiological differences in caries susceptibility. This homogeneity contrasts with the distinct subtype-specific pattern observed for dental anomalies, particularly the significantly higher burden in patients with MPS-IV. This divergence indicates that while caries risk may be broadly elevated across MPS due to common functional limitations, the development of specific dental anomalies is more closely linked to the particular skeletal and developmental pathology of each subtype.

Although we attribute the subtype-specific differences in dental anomalies primarily to the severe skeletal dysplasia characteristic of MPS-IV, alternative explanations should be considered. Variability in medical management, such as the timing and availability of enzyme replacement therapy, across subtypes may indirectly influence oral and dental development. Additionally, differences in the average age at dental examination among subtypes could affect the detection rate of certain anomalies, particularly those associated with tooth eruption and development.

The clinical implications of this study are 3-fold. First, patients with MPS-IV should be prioritized for early and comprehensive screening of dental anomalies to allow for timely orthodontic intervention. Second, all patients with MPS require intensified caries prevention strategies, with particular emphasis on pit and fissure sealing of molars. Third, those subtypes with significant neurocognitive impairment, such as MPS-III, necessitate the development of adapted oral hygiene aids and caregiver-assisted protocols. These findings support the integration of standardized oral evaluation into the multidisciplinary management framework for MPS.

Several limitations warrant careful consideration. First, the retrospective design and extraction of dental data from historical clinical records introduce potential information bias. As multiple dentists conducted examinations over the 15-year study period without formal interexaminer calibration, variations in diagnostic thresholds and recording practices are possible. To mitigate this, a standardized data extraction protocol aligned with ICDAS criteria was applied by a single primary investigator. Nevertheless, such nondifferential misclassification would likely have biased effect estimates toward the null. Second, the sample size remained limited for some subtypes after stratification, potentially reducing statistical power. Third, the absence of a healthy control group precluded direct comparison with the general population. Finally, unmeasured confounders, such as dietary habits and detailed oral hygiene practices, could not be accounted for.

Future studies should prioritize prospective, multicenter designs with precalibrated examiners to ensure diagnostic consistency. Incorporating objective biomarkers, salivary microbiological analysis, and standardized imaging protocols would help elucidate the mechanisms underlying caries susceptibility and dental anomalies in MPS. Moreover, longitudinal studies are needed to evaluate the long-term effectiveness of subtype-specific preventive and therapeutic oral health interventions.

In conclusion, this study demonstrates that Chinese pediatric patients with MPS experience a substantial burden of dental caries and anomalies, with the latter showing marked subtype-specific variation. These findings underscore the necessity of subtype-tailored oral health strategies and the integration of structured dental assessment into routine MPS follow-up care to enable early detection and intervention.

## Abbreviations

dmft: decayed, missing, and filled teeth index
GAG: glycosaminoglycan
ICDAS: International Caries Detection and Assessment System
MPS: mucopolysaccharidosis
OR: odds ratio
STROBE: Strengthening the Reporting of Observational Studies in Epidemiology

## References

[1] Nagpal R, Goyal RB, Priyadarshini K (2022). Mucopolysaccharidosis: a broad review. Indian J Ophthalmol.

[2] Bhakthaganesh K, Vanathi M, Ahmed S, Gupta N, Tandon R (2023). Mucopolysaccharidosis. Taiwan J Ophthalmol.

[3] Zhou J, Lin J, Leung WT, Wang L (2020). A basic understanding of mucopolysaccharidosis: incidence, clinical features, diagnosis, and management. Intractable Rare Dis Res.

[4] Tylki-Szymańska A, Almássy Z, Christophidou-Anastasiadou V (2022). The landscape of mucopolysaccharidosis in Southern and Eastern European countries: a survey from 19 specialistic centers. Orphanet J Rare Dis.

[5] Çelik B, Tomatsu SC, Tomatsu S, Khan SA (2021). Epidemiology of mucopolysaccharidoses update. Diagnostics (Basel).

[6] Chen X, Qiu W, Ye J, Han L, Gu X, Zhang H (2016). Demographic characteristics and distribution of lysosomal storage disorder subtypes in Eastern China. J Hum Genet.

[7] Hp C, Harjai G, Doddawad VG, S M (2023). Comprehensive preventive and therapeutic oral health care: a case report of mucopolysaccharidosis type IV A in a pediatric patient. P R Health Sci J.

[8] Carneiro NC, Abreu LG, Milagres RM (2021). Dental and maxillomandibular incidental findings in panoramic radiography among individuals with mucopolysaccharidosis: a cross-sectional study. J Appl Oral Sci.

[9] Hirst L, Mubeen S, Abou-Ameira G, Chakrapani A (2021). Mucopolysaccharidosis (MPS): review of the literature and case series of five pediatric dental patients. Clin Case Rep.

[10] de Bode CJ, Dogterom EJ, Rozeboom AV (2022). Orofacial abnormalities in mucopolysaccharidosis and mucolipidosis type II and III: a systematic review. JIMD Rep.

[11] Costi S, Caporali RF, Marino A (2022). Mucopolysaccharidosis: what pediatric rheumatologists and orthopedics need to know. Diagnostics (Basel).

[12] Oussoren E, Wagenmakers MA, Link B (2021). Hip disease in mucopolysaccharidoses and mucolipidoses: a review of mechanisms, interventions and future perspectives. Bone.

[13] Guffon N, Journeau P, Brassier A, Leger J, Chevallier B (2019). Growth impairment and limited range of joint motion in children should raise suspicion of an attenuated form of mucopolysaccharidosis: expert opinion. Eur J Pediatr.

[14] Galimberti C, Madeo A, Di Rocco M, Fiumara A (2018). Mucopolysaccharidoses: early diagnostic signs in infants and children. Ital J Pediatr.

[15] Sawamoto K, Álvarez González JV, Piechnik M (2020). Mucopolysaccharidosis IVA: diagnosis, treatment, and management. Int J Mol Sci.

[16] de Almeida-Barros RQ, de Medeiros PF, de Almeida Azevedo MQ (2018). Evaluation of oral manifestations of patients with mucopolysaccharidosis IV and VI: clinical and imaging study. Clin Oral Investig.

[17] James A, Hendriksz CJ, Addison O (2012). The oral health needs of children, adolescents and young adults affected by a mucopolysaccharide disorder. JIMD Rep.

[18] Gugnani N, Pandit IK, Srivastava N, Gupta M, Sharma M (2011). International caries detection and assessment system (ICDAS): a new concept. Int J Clin Pediatr Dent.

[19] Moradi G, Mohamadi Bolbanabad A, Moinafshar A, Adabi H, Sharafi M, Zareie B (2019). Evaluation of oral health status based on the decayed, missing and filled teeth (DMFT) index. Iran J Public Health.

[20] Talukdar L, Saha S, Dhinsa K, Rai A, Tiwari V, Trivedi H (2022). Comparison of craniofacial morphology characteristics along with dental caries status and salivary properties of operated cleft lip and palate patients with noncleft patients. J Indian Soc Pedod Prev Dent.

[21] de Santana Sarmento DJ, de Carvalho SH, Melo SL (2015). Mucopolysaccharidosis: radiographic findings in a series of 16 cases. Oral Surg Oral Med Oral Pathol Oral Radiol.

[22] Anju V, Raj NS (2024). Caries assessment and salivary microbial analysis in patients diagnosed with mucopolysaccharidosis. J Indian Soc Pedod Prev Dent.

[23] Prado HV, Carneiro NC, Perazzo MF, de Abreu MH, Martins CC, Borges-Oliveira AC (2019). Assessing a possible vulnerability to dental caries in individuals with rare genetic diseases that affect the skeletal development. Orphanet J Rare Dis.

